# Efficacy of orlistat in type 2 diabetes: a systematic review and meta-analysis

**DOI:** 10.3399/BJGPO.2025.0058

**Published:** 2025-12-19

**Authors:** Shraboni Ghosal, Neil Heron, Kayleigh Mason, Kelvin Jordan

**Affiliations:** 1 School of Health Sciences, The University of Manchester, Manchester, UK; 2 School of Medicine, Keele University, Keele, UK; 3 Centre for Public Health, Queen’s University Belfast, Belfast, UK

**Keywords:** systematic review, meta-analysis, orlistat, efficacy, type 2 diabetes, obesity

## Abstract

**Background:**

Obesity is a common comorbidity of type 2 diabetes mellitus (T2DM), a chronic metabolic condition affecting millions worldwide. Orlistat may be used to reduce weight as an adjunct to diet and lifestyle changes.

**Aim:**

To assess the evidence of orlistat on weight loss in adults with obesity and T2DM or a high risk of T2DM.

**Design & setting:**

Systematic review and meta-analysis of randomised controlled trials (RCTs) in clinical settings.

**Method:**

Articles were searched in 10 databases including MEDLINE, Embase, and PsycInfo. RCTs of orlistat in adults with T2DM or at high risk and a body mass index (BMI)≥25 kg/m^2^, with ≥12 weeks of follow-up and reported change in weight or BMI, were included. A random effects meta-analysis model was used to pool mean differences, *I* ^*2*^ statistics to assess heterogeneity, and a funnel plot to assess publication bias.

**Results:**

Thirty RCTs compared orlistat with a comparator, in conjunction with a weight-loss diet. All trials showed statistically significant (*P*<0.05) greater weight loss for the orlistat group than controls. A meta-analysis of 22 studies (*n* = 5921) showed that the overall weight loss for the orlistat group was a mean 2.40 kg (95% confidence interval [CI] = 2.08 to 2.72) greater than in controls. Weight difference was statistically significant (*P*<0.05) between subgroups at 3 months (2.74 kg, 95% CI = 1.20 to 4.27), 6 months (2.13 kg, 95% CI = 1.61 to 2.66), and 12 months (2.49 kg, 95% CI = 1.89 to 3.09).

**Conclusion:**

Orlistat resulted in statistically significant greater weight loss in overweight adults with T2DM or at high risk compared with controls. Orlistat can be considered an adjunct in T2DM or at high risk of T2DM for weight loss along with diet and lifestyle modifications.

## How this fits in

Obesity is a risk factor for developing type 2 diabetes mellitus (T2DM) in those at high risk; however, previous systematic reviews have assessed weight loss mainly in obesity or T2DM. Recent systematic reviews quantifying the magnitude of weight loss with orlistat, a lipase inhibitor, in T2DM and those at high risk of T2DM, are lacking. This systematic review and meta-analysis adds to the research as it suggests that adults with T2DM or at high risk may lose on average 2.40 kg more weight with orlistat at 12 months of follow-up. Orlistat can be considered a useful adjunct to help adults with T2DM or at high risk to lose weight along with diet or lifestyle modifications but may not be tolerated by all in the long term.

## Introduction

Type 2 diabetes mellitus (T2DM) is a chronic condition and a major global public health issue, and remains a world health challenge.^1^ Worldwide, 537 million people are estimated to have diabetes, which is predicted to rise to 643 million by 2030 and to 783 million by 2045.^2^ In the UK, approximately 90% of adults with diabetes have T2DM.^3^ Prediabetes is also a growing public health problem and 541 million adults are at high risk of diabetes globally.^2^ Those with prediabetes or impaired glucose tolerance (IGT) have an increased risk of developing T2DM with 5%–10% progressing to T2DM every year.^4^ Diabetes may lead to an increased risk for cardiovascular disease (CVD), retinopathy, neuropathy, kidney disease, and premature mortality.^5^

Obesity increases the risk of many conditions including T2DM.^3^ In the UK, 90% of adults with T2DM are overweight or have obesity.^5^ Diabetes-related complications can cause disability, reduce quality of life (QoL), and increase the risk of death.^2^ In T2DM, insulin resistance (IR) and dysregulated glucose metabolism may contribute to weight gain.^6^ Obesity is the most important modifiable risk factor for T2DM and prediabetes, and weight loss is an important therapeutic strategy.^3,5^ Weight loss can reduce blood glucose levels and IR, and improve insulin sensitivity.^5^ A weight loss of 5%–10% or more is a recommended aim in those with a body mass index (BMI) of ≥35 kg/m^2^.^3^

Lifestyle modifications are recommended for the initial treatment of obesity.^3^ However, weight loss and weight management can be challenging for people with T2DM or prediabetes.^7^ Lifestyle or behavioural interventions are often less effective than pharmacotherapy for weight loss in T2DM owing to the challenges of adherence, modest outcomes, and inability to influence physiological mechanisms directly.^6,8^ Therefore, additional interventions, such as pharmacotherapy, may be necessary for weight management in T2DM. Weight-reducing medications, such as orlistat (tetrahydrolipstatin), a lipase inhibitor, have been suggested as an adjunct to diet and lifestyle changes in adults with overweight or obesity and T2DM or prediabetes.^3^ Orlistat, by inhibiting fat absorption, may offer a more structured approach to weight loss in T2DM and also lead to improved blood glucose levels.^8^

Orlistat is a National Institute for Health and Care Excellence (NICE)-approved oral treatment for obesity in the UK, with a licence to be prescribed as an adjunct to diet and exercise, for people with a BMI of≥30 kg/m^2^ or a BMI of≥28 kg/m^2^ with risk factors such as T2DM.^9,10^ Orlistat (as are some glucagon-like peptide-1 receptor agonists [GLP1-RAs] such as liraglutide and semaglutide) is available through the NHS in the UK for obesity treatment.^11,12^ However, liraglutide and semaglutide are injectable and can be expensive, whereas orlistat is an oral medication, with some evidence of cost-effectiveness.^13^ In a recent retrospective, longitudinal UK cohort study, orlistat was found to be associated with modest and sustained weight loss that also improved glycaemic and metabolic parameters in T2DM and prediabetes.^14^

Several systematic reviews in the past have highlighted the efficacy of orlistat for weight loss in the general population.^15–17^ Orlistat, along with lifestyle changes, can lead to greater weight loss than lifestyle changes alone^18^ and also reduce the risk of developing T2DM.^19^ Orlistat use has been associated with improved glycaemic control and reduced blood pressure (BP) in the long term.^20^ However, previous randomised controlled trials (RCTs) were limited with small sample sizes and a lack of statistical power.^21^ While studies have examined weight loss with orlistat, they were conducted mainly in the general population of those with obesity or overweight, and there has been no recent systematic review of the evidence for weight loss in T2DM or prediabetes. The aim of this systematic review and meta-analysis was to investigate the effect of orlistat on weight loss in adults who are overweight and obese with T2DM, prediabetes, or at high risk of developing T2DM, using evidence from RCTs.

## Method

### Search methods and data sources

A systematic search of the scientific literature was conducted. A base strategy was developed in the Ovid and EBSCO (Elton B. Stephens Company) interfaces, which provide access to a range of bibliographic databases (see Supplementary File 1). This was converted to run in other databases effectively using different interfaces. Ten online bibliographic databases were searched: the Cochrane Library, MEDLINE (Medical Literature Analysis and Retrieval System Online), CINAHL Plus (Cumulative Index of Nursing and Allied Health Literature), Embase (Excerpta Medica Database), PsycInfo, AMED (Allied and Complementary Medicine), (DARE) Database of Abstracts of Reviews of Effects, Google Scholar, PubMed, and Web of Science (see Supplementary Table 1). Hand-searches were also conducted and reference lists of identified articles were examined. The full texts of relevant articles were retrieved and full-text review was undertaken for any title or abstract that did not clearly meet the exclusion criteria. The protocol was registered with PROSPERO (CRD42019147972) as part of a larger review. For transparency and reproducibility, a full description of the search terms and methodology for all the databases has been provided in Supplementary Table 1.

### Inclusion criteria

RCTs of ≥12 weeks in duration published in English between 1 January 1998 and 20 June 2024 were eligible for review. The eligibility criteria are summarised using Population, Intervention, Comparator and Outcome (PICO):

Population: adults aged≥18 years with BMI≥25 kg/m^2^ with T2DM, prediabetes, or at high risk of T2DM.

Intervention: orlistat alone or combined with lifestyle factors (diet and/or exercise) or other weight-loss strategies such as behavioural or educational factors.

Comparator: placebo, lifestyle factors (diet and/or exercise), medications, for example, metformin or usual care (UC) by healthcare professionals (HCPs). People may also have received treatment for glycaemic control (for example, metformin, sulphonylurea, insulin, or none).

Primary outcome: weight loss (difference between baseline and follow-up weight measures).

Secondary outcomes: changes in BMI, measures of glycaemic control (glycosylated haemoglobin [HbA1c], fasting plasma glucose [FPG]), blood pressure (BP), quality of life (QoL), and side effects.

### Exclusion criteria

Observational studies, guidelines, editorials, quasi-randomised trials, patient populations with uncontrolled hypertension, chronic heart or renal disease, hypothyroidism, polycystic ovarian syndrome, only obesity and without T2DM or prediabetes or at high risk of T2DM (in subgroups), previous bariatric surgery, pregnancy or lactating persons, relevant psychiatric or medical illness and considerable weight loss before screening, were excluded. Conference proceedings, dissertations, commentaries, protocols, and letters were also excluded.

### Study selection

The lead author (SG) conducted the review, screened titles, abstracts, and articles for eligibility, and repeated the process at a later time point for reliability purposes. All citations from electronic database searches were imported into an Excel spreadsheet for selecting articles that met the inclusion criteria. The study selection process is presented in a Preferred Reporting Items for Systematic Reviews and Meta-Analyses (PRISMA) flowchart.^22^ An attempt was made to contact study authors to provide the full text when it was not available. The search strategies are provided in Supplementary Table 1.

### Data extraction

Data were extracted from trials and reported according to Preferred Reporting Items for Systematic reviews and Meta-Analyses (PRISMA) guidelines.^22^ A matrix table of study characteristics was produced, along with a descriptive summary of individual papers. The data extracted included the author or authors, year of publication, location or country, design, duration, sample size, details of orlistat intervention, mean age or age range, sex distribution, follow-up duration, changes in weight, and secondary outcomes (see Supplementary Tables 2 and 3).

### Risk of bias and quality assessment

Quality assessment was undertaken using the Critical Appraisal Skills Programme (CASP)^23^ tools. The methodological quality of papers was assessed using the Jadad quality score^24^ (see Supplementary Table 1). This tool allocates RCTs a score of between 0 (very poor) and 5 (rigorous) points.^24^ RCTs were assessed in terms of domains such as attrition bias, detection bias, performance bias, reporting bias, selection bias, and other bias. The quality assessment tool used (CASP), along with a detailed description of the individual quality assessment points, has been provided in Supplementary Tables 4 and 5.

### Statistical analysis

A meta-analysis of change in weight was undertaken using Review Manager (RevMan, 5.4.1, Cochrane Collaboration, 2020)^25^ for studies sufficiently similar in design, comparator group, length of follow-up (that is, 3 months, 6 months, and 12 months), and the outcome measurement of change in weight. We only included trials that reported, or could be converted to, metric unit for weight change (mean difference in kg ± standard deviation [SD]) and so excluded from the meta-analysis those, for example, which only looked at percentage change. A sensitivity analysis was conducted by selecting trials that were deemed not at risk of bias.

We assessed the assumption of homogeneity of true effect sizes and calculated the degree of inconsistency across studies. Heterogeneity between study estimates was incorporated using a random-effects model.^26^

Data were pooled using the DerSimonian and Laird formula for calculating between‐study variance.^27^ The *I^2^* statistic determines the proportion of variance explained by between-study heterogeneity.^28^ An *I^2^* value of zero indicates no heterogeneity and values of 25%, 50%, and 75% indicate low, moderate, and high heterogeneity, respectively.^28^ Bias in meta-analysis can be detected by a simple, graphical test.^29^ To determine publication bias related to the meta-analysis, a funnel plot was created as it is a standard method to assess the risk of bias by visual inspection.^29^ Funnel plots of the RCTs’ effect estimates against sample size can be skewed and asymmetrical in the presence of publication bias and other biases.^29^

## Results

A flow diagram summarises the systematic search and study selection process and includes results of the search (Supplementary Figure 1). The initial search identified a total of 12 352 articles with 30 RCTs matching the inclusion criteria and included in the review.

### Study characteristics

The characteristics of the 30 RCTs are presented in Supplementary Table 2. Study follow-up ranged from 12 weeks^30–33^ to 4 years^34^ with different diagnostic criteria used to classify T2DM, prediabetes, and those at high risk. There were a total of 11 208 participants included in the trials. Twenty-four studies were in a population with T2DM and the others were in prediabetes or at high risk of T2DM or both. All studies were conducted between 1998 and 2017, with most RCTs receiving funding from the medication manufacturer. The number of participants in trials ranged from 29–3305 and participants had a BMI of ≥25 kg/m^2^. Studies were conducted in the US (*n* = 7), Europe (*n* = 16), Brazil (*n* = 1), China (*n* = 1), Bangladesh (*n* = 1), Turkey (*n* = 1), Taiwan (*n* = 1), Canada (*n* = 1), and New Zealand (*n* = 1). All 30 studies were RCTs,^30–59^ in which orlistat was used in conjunction with a weight-loss diet. All RCTs had dispensed orlistat to participants at a standard dosage of 120 mg three times per day (see Supplementary Table 2). All trials included a standardised hypocaloric diet for the treatment and/or comparator groups as well as behavioural or dietary counselling by HCPs, with or without physical activity.

### Weight loss with orlistat

The RCTs included in this systematic review reported overall mean weight change from 3 months to 4 years of follow-up, which was consistently higher in the orlistat group.^30–59^ Weight loss was significantly more in the orlistat groups compared with placebo (*P*<0.05), irrespective of whether they had received antidiabetic treatments such as, metformin,^30,35,37,41,44,49^ sulphonylurea,^35–37,41,44,49^ or insulin.^33,37,49^ In RCTs examining weight loss as a percentage of baseline weight, more people in the orlistat groups (120 mg, thrice per day) compared with controls, lost ≥5% of baseline weight (30%–65.7% versus 7%–43.6%) in 12 RCTs^30,34–41,44,48,54^ and ≥10% of baseline weight in 11 trials (10%–63.0% versus 3.9%–24.8%; all *P*<0.05).^34–38,41,44,45,48,54,55^ After 6 months of treatment, the orlistat group showed significantly reduced weight, for example, by about ≥6% in four trials^36,40,43,51^ and 10% in another trial.^42^

In RCT with weight-loss maintenance phases, there was less weight regain in the orlistat group than controls.^36,48,53^ The RCTs showed that weight loss was generally maintained in adults who continued orlistat. Trials showed that after 2 years of orlistat treatment, 57% patients maintained ≥5% weight loss^55^ and 34.1% maintained a weight loss of≥10% compared with placebo.^48^ Although not outcomes specified for this review, there was a decreased risk of incident T2DM in people at high risk^34,48,53^ and a reduction of antidiabetic medications in four trials.^34,48,53,59^ T2DM development reduced by 37.3% in the orlistat group,^34^ and in another trial, more people in the placebo group developed T2DM compared with the orlistat group (11% versus 5%).^53^

### Meta-analysis of change in weight

A meta-analysis of change in weight was also undertaken. The RCTs of orlistat in the adult T2DM and prediabetes or high-risk populations reviewed here (*n* = 5921) were stratified by duration of follow-up, and only the trials that demonstrated sufficient similarity in design and had the same period of follow-up (that is, 3 months, 6 months, or 1 year) in the reporting of data (*n* = 22) were included. A forest plot for all 22 estimates of change in weight stratified by follow-up period is shown in Supplementary Figure 2. There was high heterogeneity between the studies (*I^2^* = 83%; *P*<0.001); however, all 22 studies showed more reduced weight at 3 months, 6 months, and 12 months in the orlistat group compared with controls, although the magnitude of this effect varied between studies (see Supplementary Figure 2).

Across the 22 studies, the overall pooled mean difference for change in weight was 2.40 kg (95% confidence interval [CI] = 2.08 to 2.72). The pooled mean difference for change in weight at 3 months was 2.74 kg (95% CI = 1.20 to 4.27), at 6 months was 2.13 kg (95% CI = 1.61 to 2.66), and at 12 months was 2.49 kg (95% CI = 1.89 to 3.09) (see Supplementary Figure 2). Funnel plot symmetry was examined in the 22 studies included in the meta-analysis to assess publication bias, as demonstrated in Supplementary Figure 3. A sensitivity analysis was also conducted, however, all these effects were consistent and robust and there was no significant change in our findings (see Supplementary Figures 4 and 5).

### Glycaemic parameters: HbA1c and/or fasting plasma glucose

Twenty-nine out of 30 RCTs reported the effects of orlistat on glycaemic parameters (HbA1c and/or fasting plasma glucose [FPG]) in addition to weight or BMI^30–56,58,59^ (see Supplementary Table 3). Orlistat groups showed statistically significant (*P*<0.05) decreases in glycaemic control (FPG and/or HbA1c levels), along with reduced IR, and improved insulin sensitivity in the trials. Orlistat significantly reduced HbA1c and/or FPG concentrations compared with controls (*P*<0.05) in the trials, and this was consistent over follow-ups of 3 months,^30–33,49^ 6 months,^38,39,51,52^ 12 months,^35,36,40,41,47,49,50^ 2 years,^55^ and 3 years.^53^ RCTs reported that orlistat improved glycaemic control sooner than controls without any serious unpleasant events.^30–33,35,38,40,41,43,46–49,51–53^ However, one RCT with the lowest change in HbA1c found no significant difference in HbA1c between orlistat group and controls at 12 weeks.^45^ Most studies reported that FPG levels also reduced significantly (*P*<0.05).^32–36,38–41,43,46–49,51–53,55^ In RCTs over 12 months with T2DM adults with obesity, orlistat also reduced weight or BMI and IR in addition to improving glycaemic parameters and insulin sensitivity, versus placebo.^43,47,49,50^

### Orlistat on blood pressure and quality of life

Orlistat’s effect on BP was reported by trials and greater reductions in BP were observed than in the control group.^31–33,37,38,41,47,48,51,52,59^ Significant systolic blood pressure (SBP) and/or diastolic blood pressure (DBP) improvements (*P*<0.05) in the orlistat group after 3 months, 6 months, or 12 months or 2–3 years were observed in RCTs^31–33,35,38,41,44,47,48,51–53,55^ (see Supplementary Table 3). In a 1-year trial, although significant changes were observed in many individual CVD risk factors in the orlistat group, no difference was found in the change in 10-year CVD risk between orlistat and placebo groups.^59^ However, in another trial, the estimated 10-year CVD risk was found to reduce significantly (*P*<0.05) in the orlistat group.^51^ QoL was measured in only one trial using the Short Form Health Survey (SF-36) questionnaire.^59^ Improvements were seen in the ‘vitality’ and ‘physical functioning’ domains; however, ‘vitality’ was the only domain that had a statistically significant (p<0.006) difference between the orlistat and placebo groups, with the orlistat group having a higher (better) score at 52 weeks.^59^

### Attrition rates, and adverse and gastrointestinal events

Out of trials reporting attrition (20 of the 30 trials), in the orlistat groups, attrition rates ranged from 0%–81%. A higher number of adverse events were reported in the orlistat group compared with placebo; however, in five trials where attrition rates were reported, the control group had a higher attrition rate than the orlistat group^35–37,53,55^ (see Supplementary Table 3). The most common reasons reported for stopping orlistat were refusing to have treatment, loss to follow-up, and adverse effects.^30,34–39,41,42,44,47,48,53,54,56,57,59^ Trials reported increased gastrointestinal (GI) side effects with orlistat than in controls.^30,35–37,39,40,42,44,48,53,54^ High dropout rates were reported in a few trials with orlistat,^35,59^ with 57% in the 4-year XENDOS study.^34^ However, others reported that attrition rates in the orlistat group compared with controls were none,^32^ low,^35–38,55,59^ or similar in both groups.^30,32,34,39,44,54^ Most RCTs reported that orlistat was well tolerated, with no serious adverse effects,^31,32,34,36,38,41–43,47,49–51,56^ while others reported a few serious adverse events in the orlistat group.^30,48,51,56^ Withdrawals from the orlistat group were mostly owing to GI events in one RCT;^59^ however, in another, adverse events were lower in the second year than in the first.^48^ However, these events were mostly unrelated to orlistat and no deaths were reported.^30,34,56^ Orlistat also did not have associations with any cardiovascular (CV) events.^47,49,50^

### Risk of bias and quality assessment

The methodological qualities of included studies were good (see Supplementary Table 2). Studies had reported clearly defined aims and objectives, appropriate study designs, selection of participants, standardised data collection methods, measurements and analysis of outcomes. Risk of bias was low in all included studies (see Supplementary Tables 4 and 5). Out of the 30 RCTs, there were 27 double-blind and three open-labelled trials.^31,51,52^ Most studies included a single blind pre-treatment phase, of 2 weeks,^30,37–39,44,49,57^ 4 weeks,^40,48,58^ 5 weeks,^36,55^ 8 weeks,^53^ or 12 weeks.^31^ Trials with orlistat used the ‘intention-to-treat’ method of analysis except two.^46,48^ All orlistat groups in the included RCTs had also received hypocaloric diets and/or dietary or lifestyle counselling by HCPs.

A quality scoring tool by Jadad *et al* ^24^ as well as CASP tools^23^ were used to assess quality of the studies. The CASP tools showed that the RCTs were mostly of similar quality with low risk of bias (see Supplementary Table 4). However, one methodological limitation was the attrition rates, which varied between studies and were not reported in some trials (see Supplementary Table 3). Overall, all included studies that underwent quality assessment with the use of Jadad scale, received a total of 3–5 points, and were thus deemed to have a low risk of bias (see Supplementary Table 2). Funnel plot symmetry was examined in the 16 studies included in the meta-analysis to assess publication bias, demonstrated in Supplementary Figure 3. The risk of bias was assessed in the meta-analysis through the funnel plot by visual inspection.^29^ On visual examination of the funnel plot, the number of studies at the left and right side of the funnel plot was roughly the same, showing that the plot was symmetrical, indicating no evidence of publication bias.

### Sensitivity analysis

For evaluating the reliability of our meta-analysis, a sensitivity analysis was conducted (*n* = 2871) by excluding 11 studies deemed at a high risk of bias. However, our findings showed no significant difference (see Supplementary Figures 4 and 5). Our sensitivity analysis also did not lead to any significant change in the interpretation of evidence, indicating that the meta-analysis had good reliability.

## Discussion

### Summary

This systematic review assessed RCTs that evaluated orlistat’s effects on weight loss in adults who are overweight or obese with T2DM or high risk of T2DM. All trials compared orlistat with placebo and reported a statistically significant greater weight loss in the orlistat group. This review showed a consistently greater weight loss in the orlistat group, with all 30 trials showing greater weight loss over follow-up periods from 3 months to 4 years. Orlistat also showed significant decreases in glycaemic control (FPG and/or HbA1c levels), reduced IR, and improved insulin sensitivity.^30–59^ Although not outcomes for this review, there was a decreased risk of incident T2DM in people at high risk^34,48,53^ as well as reduction in antidiabetic medications,^34,48,53,59^ as shown in some trials.

Our meta-analysis showed an overall mean weight loss of 2.40 kg with orlistat compared to placebo and also in the subgroup analyses. Maunder *et al* ^60^ emphasised a difference of ≥2.50 kg may be considered as clinically significant. RCTs that reported weight loss in relative terms, reported a statistically significant greater percentage of people with a weight loss of ≥5% for orlistat than in control groups, consistent with NICE guidelines.^61^ However, all orlistat groups in the included trials had also received hypocaloric diets and/or dietary or lifestyle counselling by HCPs, which need to be taken into consideration.

The *I^2^* values indicated high heterogeneity across all studies for each outcome, likely to have stemmed from variations in interventions, follow-up durations, and HCPs involved. The findings of this review suggest that orlistat can help people with T2DM achieve weight loss more than only by dietary changes in adults with overweight or obesity, which was maintained in those who continued orlistat. However, orlistat is not always well tolerated and may be stopped early.^14^ Our review suggests that weight-reducing medications, such as orlistat, still have a place and can be used as an adjunct to diet and lifestyle changes for the management of overweight or obesity in adults with T2DM or prediabetes or at high risk of T2DM.

### Strengths and limitations

This systematic review has several strengths with a focus on people with T2DM or prediabetes or at high risk of T2DM and who are overweight or obese. A robust search strategy was systematically applied along with a study selection strategy, capturing all recent RCTs. A meta-analysis with 22 studies was also used to assess change in weight with orlistat and placebo in those with T2DM or at high risk of T2DM. A strength of our review is that it includes the number of RCTs that were included in this meta-analysis, which also produced no changes in the sensitivity analysis for interpreting the evidence (see Supplementary Figures 4 and 5). A valid quality scoring tool by Jadad *et al* ^24^ for assessing quality of the studies and the addition of CASP tools^23^ to critically appraise each RCT were used (see Supplementary File 1, Tables 2 and 3).

Our findings regarding weight loss effects of orlistat are consistent with results from previous systematic reviews that there was significantly greater weight loss in the orlistat groups compared with placebo. Our review may also be relevant for policy and clinical practice. This review suggests orlistat is effective for weight loss in individuals with T2DM or prediabetes and obesity or overweight but its use should be balanced against potential side effects. While NICE guidelines recommend orlistat for obesity treatment, decisionmaking around prescribing it to take into account individual patient needs and preferences. Further evidence of its effectiveness and patient acceptability and adherence (given its side effects) from real-world practice would also be useful.

The limitations of this review are that the sample sizes of some of the studies were low, and possible overestimation of orlistat’s effects in the longer term given most evidence were from interventions conducted over a shorter term (that is, up to 6 months or 12 months). Eight trials were not included in the meta-analysis owing to differences in trial protocols or differences in reporting. The meta-analysis showed marked heterogeneity with regard to weight-loss estimates, while studies varied in demographic characteristics of study participants and length of follow-up. Additionally, most of the RCTs had narrow inclusion criteria and the treatment strategies included changes in diet and some oral antidiabetic medications alongside orlistat.

Some differences between controls and interventions were noted in a few trials and QoL was inconsistently reported. The other limitations of this review included exclusion of unpublished studies or those in languages other than English, absence of grey literature, and searches and data extraction were conducted by only one reviewer, which may cause validity or reliability issues. However, 10 databases were searched, the search strategy was robust and followed the PRISMA guidelines, and hand searching was also conducted.

There was a lack of blinding in three trials, which may have introduced biases, and another limitation was sponsorship bias in most trials, owing to funding by medication manufacturers. We also cannot rule out the effect of additional dietary and lifestyle factors and/or advice by HCPs. A further potential limitation is that all orlistat groups in the included RCTs had also received hypocaloric diets and/or dietary or lifestyle counselling by HCPs, meaning that additional diet or lifestyle changes were potential confounders of the observed effect. However, orlistat is recommended as an adjunct in obesity, in conjunction with a mildly hypocaloric diet.^3^

### Comparison with existing literature

Findings from this review are consistent with findings from previous systematic reviews regarding obesity management with orlistat.^15–17,62,63^ However, our study extends these reviews by providing an updated assessment of literature and focusing specifically on T2DM, prediabetes, and high risk. Supported by sub-group meta-analysis, we elucidated orlistat’s efficacy in T2DM and prediabetes by duration of follow-up. In a previous meta-analysis, Norris *et al* ^63^ showed a reduction of 2.6 kg (95% CI = 2.1 to 3.2) with orlistat in T2DM, similar to our findings. Others also showed similar weight losses of 2.12 kg (95% CI = −2.51 to −1.74)^16^ and 2.7 kg (95% CI = 2.3 to 3.1)^62^ in previous systematic reviews; however, these were conducted in the general population.

Our subgroup analysis showed statistically significant weight losses at 3 months, 6 months, and 12 months with orlistat in those with T2DM, prediabetes, and high risk. A weight loss of ≥5% may reduce disease risk along with metabolic improvements.^51^ Although a≥5% weight loss in 12 weeks is usually considered clinically meaningful,^3^ less strict goals apply in those with T2DM.^12^ Previous reviews of RCTs in the general population have reported that the mean attrition rate for orlistat was 33%^62^ and higher.^64^ Similarly, a population-based cohort study also reported high discontinuation rates.^65^ However, any weight loss or decreases in glycaemic parameters and systolic BP can be beneficial in adults with T2DM or prediabetes and obesity, as they can reduce potential for complications,^37,43,46–48,50,52^ and also prevent future CV events.

### Implications for research and practice

The findings from this systematic review support the use of orlistat in conjunction with a hypocaloric diet or lifestyle changes for managing obesity in T2DM and prediabetes or IGT. Weight loss is fundamental to the management of T2DM or prediabetes, in those with overweight or obesity. In people with T2DM or prediabetes and obesity or overweight, weight loss of 5%–10% can improve glycaemic control and insulin sensitivity, and reduce IR and CVD risk factors, and the need for antidiabetic medications.^3,9,12,43,61^ However, long-term weight management can be more difficult in T2DM.^6,7^ Orlistat, when combined with a low-fat diet, can be an effective option for achieving clinically meaningful weight loss.^64^

Orlistat may also not be tolerated by everyone and can be stopped early or if <5% weight loss is achieved in 12 weeks.^66^ Orlistat is known to cause some side effects, for example, GI disorders, nausea, fatty stools, faecal urgency, flatulence, abdominal pain, and a decrease in fat-soluble vitamins.^12,66^ Despite this, orlistat can be considered in those with T2DM or prediabetes or those at high risk of T2DM and with obesity or overweight, as the side effects of orlistat can often be reduced by limiting fat intake.^12^ Orlistat is also contraindicated in those with cholestasis or chronic malabsorption syndrome, and caution should be also taken in those with chronic kidney disease (CKD).^67^ However, the European Medicines Agency (EMA) reviewed orlistat’s safety concerns in 2012 and concluded that the benefits of orlistat outweigh the risks.^68^ Weight regain has been observed following withdrawal of orlistat^69^ although this was mainly owing to non-compliance.^70^

Orlistat had been associated with 3.1% greater weight loss than placebo; however, this was considered to be modest when compared with greater weight losses observed with some of the GLP1-RAs.^69^ Despite the modest weight loss, orlistat remains a cost-effective, oral treatment,^13^ which is also available over the counter at a lower dosage (that is, 60 mg), while the GLP1-RAs, such as liraglutide, semaglutide, and tirzepatide, are injectable and can be expensive.^67^ These T2DM medications have shown promise for weight loss; however, the potential risks with some of them, for example, renal impairment, pancreatitis, heart failure, medullary thyroid carcinoma, or psychiatric adverse events, are concerning.^71,72^

Orlistat, along with behavioural and lifestyle interventions, still has a place as an effective anti-obesity medication, as weight management is an essential component in T2DM and prediabetes. Orlistat may also benefit glycaemic control (that is, decrease FPG and HbA1c levels) as well as BP. However, further research is needed on these outcomes and adverse events such as GI side effects, and other contraindications. The prevention of weight regain after initial weight loss is an important aspect of any weight management strategy and this review suggests that weight loss was generally maintained in those who continued orlistat, but longer-term studies are needed. These findings are important for developing effective policies and practice.

The effectiveness of orlistat in subgroups (for example, based on baseline weight, age, sex, and comorbid conditions such as hypertension) should also be a focus for further research. Orlistat, taken with an appropriate diet, promotes clinically significant weight loss and in one RCT, it reduced weight regain in patients with obesity over a 2-year period.^55^ However, the use of orlistat beyond 2 years needs careful monitoring with respect to efficacy and adverse events, and needs further research.

In conclusion, orlistat in conjunction with a hypocaloric diet and lifestyle modification can be advantageous for people with T2DM or prediabetes and obesity or overweight, with modest but potentially important reductions in weight up to 12 months following treatment initiation. However, there is the potential of poor compliance owing to side effects, and safety aspects over the long term also remain unclear. Future work investigating whether hypertension or glycaemic control improves in both T2DM and prediabetes following treatment with orlistat and over the long term is required. Impact of dosage and long-term safety aspects also warrant further investigation.

## Protocol registration number

PROSPERO: CRD42019147972.
